# A meta-analysis of photosynthetic efficiency and stress mitigation by melatonin in enhancing wheat tolerance

**DOI:** 10.1186/s12870-024-05132-2

**Published:** 2024-05-21

**Authors:** Ihsan Muhammad, Fahim Ullah, Shakeel Ahmad, Bandar M. AlMunqedhi, Dunia A Al Farraj, Mohamed S Elshikh, Weijun Shen

**Affiliations:** 1 Guangxi Key Laboratory of Forest Ecology and Conservation, State Key Laboratory for Conservation and Utilization of Agro-bioresources, College of Forestry, Guangxi University, 100 Daxue Rd., Xixiangtang District, Nanning, Guangxi 530004 China; 2 Department of Plant Breading and Genetics, The University of Agriculture Swat, Swat, Pakistan; 3 State Key Laboratory for Conservation and Utilization of Subtropical Agro-bioresources, College of Life Science and Technology, Guangxi University, Nanning, China; 4 Department of Botany and Microbiology, College of Science, King Saud University, P.O. 2455, Riyadh, 11451 Saudi Arabia

**Keywords:** Photosynthetic rate, Fluorescence yield, Transpiration rate, Abiotic stress, Stomatal conductance, Melatonin

## Abstract

**Background:**

Our meta-analysis examines the effects of melatonin on wheat under varying abiotic stress conditions, focusing on photosynthetic parameters, chlorophyll fluorescence, leaf water status, and photosynthetic pigments. We initially collected 177 publications addressing the impact of melatonin on wheat. After meticulous screening, 31 published studies were selected, encompassing 170 observations on photosynthetic parameters, 73 on chlorophyll fluorescence, 65 on leaf water status, 240 on photosynthetic pigments.

**Results:**

The analysis revealed significant heterogeneity across studies (I² > 99.90%) for the aforementioned parameters and evidence of publication bias, emphasizing the complex interaction between melatonin application and plant physiological responses. Melatonin enhanced the overall response ratio (ln*RR*) for photosynthetic rates, stomatal conductance, transpiration rates, and fluorescence yields by 20.49, 22.39, 30.96, and 1.09%, respectively, compared to the control (no melatonin). The most notable effects were under controlled environmental conditions. Moreover, melatonin significantly improved leaf water content and reduced water potential, particularly under hydroponic conditions and varied abiotic stresses, highlighting its role in mitigating water stress. The analysis also revealed increases in chlorophyll pigments with soil drenching and foliar spray, and these were considered the effective application methods. Furthermore, melatonin influenced chlorophyll SPAD and intercellular CO_2_ concentrations, suggesting its capacity to optimize photosynthetic efficiency.

**Conclusions:**

This synthesis of meta-analysis confirms that melatonin significantly enhances wheat’s resilience to abiotic stress by improving photosynthetic parameters, chlorophyll fluorescence, leaf water status, and photosynthetic pigments. Despite observed heterogeneity and publication bias, the consistent beneficial effects of melatonin, particularly under controlled conditions with specific application methods e.g. soil drenching and foliar spray, demonstrate its utility as a plant growth regulator for stress management. These findings encourage focused research and application strategies to maximize the benefits of melatonin in wheat farming, and thus contributing to sustainable agricultural practices.

**Supplementary Information:**

The online version contains supplementary material available at 10.1186/s12870-024-05132-2.

## Introduction

Wheat, synthetically known as *Triticum aestivum* L., is a crucial cereal grain integral to the global food supply, providing essential nutrients for humans nutrition [1]. Its straw is also used as livestock feed, and it is used in the manufacturing of cardboard, hardboard, and other industrial products [2]. As a major source of carbohydrates, vitamins, proteins, and minerals, wheat has evolved through selective breeding, resulting in numerous varieties adapted to various climatic conditions and soil types. Bread wheat, also known as *Triticum aestivum*, is renowned for its high gluten content, which is crucial for the texture and structure of bread [3]. Additionally, wheat is essential in producing pasta, spaghetti, macaroni, semolina, couscous, and unleavened bread, largely due to its high protein content [4, 5]. Technological advances in agriculture have led to the development of high-yielding and disease-resistant wheat cultivars, significantly enhancing grain production and addressing food scarcity [6, 7]. Wheat plays a significant role in global markets, affecting the economies of major producing countries like Russia, the United States, and China [8].

Various abiotic variables such as drought, salinity, heavy metals, waterlogging, infrared radiation, and extreme temperatures often pose significant threats to wheat production [9–11]. These stressors severely impact plant growth, development, and yield of staple crops such as wheat, maize, rice, and barley, highlighting a major challenge in agricultural production [12]. In the context of global climate change, understanding and mitigating the adverse effects of these stresses is crucial. Wheat, a widely cultivated and consumed grain, is particularly affected by these conditions. Abiotic stressors dramatically reduce photosynthetic parameters, chlorophyll fluorescence, alter leaf water status, and disrupt photosynthetic pigments, and affect chlorophyll. Drought stress leads to water scarcity, impairing photosynthesis and nutrient uptake, which results in stunted growth and physiological abnormalities that reduce crop production [11, 13, 14]. Additionally, drought stress triggers cellular dehydration, oxidative stress, and metabolic disruptions, necessitating complex plant regulatory responses. Previous studies demonstrated that drought stress and boron toxicity significantly reduce wheat’s chlorophyll content, essential for photosynthesis, thereby diminishing photosynthetic efficiency and growth [15–17].

Reduced chlorophyll levels directly impact photosynthetic capacity [18]. Abiotic stressors also affect plant hydration altering cell turgidity and water status, which in turn affects light quenching mechanisms as plants attempt to dissipate excess energy under stress to protect their photosynthetic systems. Similarly, waterlogging stress affects physiological and biochemical traits in maize, potentially applicable to wheat [19]. Gas exchange parameters such as photosynthetic rate, stomatal conductance, and transpiration rate for wheat are significantly influenced under abiotic stress [20]. Salinity results from poor drainage and evaporation, causing surface salt accumulation in irrigated lands [21–23], leading to ion toxicity, osmotic imbalance, and nutritional disparities. These conditions severely affect metabolic processes, including glucose and lipid metabolism and protein synthesis [24]. Moreover, the escalating issue of heavy metals pollution in soil from contaminants like Cd, Cr, and Al is a growing anxiety. These metals are highly toxic, including causing oxidative stress, enzyme inhibition, and cellular damage [11, 25]. Extreme temperatures exceeding optimal growth levels cause protein denaturation, cell membrane instability, and increased reactive oxygen species (ROS) production [26]. Conversely, cold stress impacts cell membrane flexibility, enzyme function, and results in ice crystal formation within cells [27].

Melatonin, chemically known as N-acetyl-5-methoxytryptamine, is a naturally occurring hormone found in animals, plants, fungi, and bacteria. In humans and animals, it regulates sleep-wake cycles and other physiological functions. In plants, melatonin acts as a growth regulator and an adaptive response mediator to environmental stresses. Recent studies have highlighted melatonin’s efficacy in mitigating abiotic stresses in wheat [26, 28, 29]. As a growth regulator and antioxidant, melatonin enhances tolerance to various abiotic stresses by enhancing antioxidant defense systems and regulating hormonal balances in wheat [30]. It positively influences transpiration rate, stomatal conductance, photosynthetic rate, leaf water potential, chlorophyll SPAD, water use efficiency [31], enhances plant resilience and crop yield by bolstering the antioxidant defense system [32], and increases plant resilience to abiotic stress [33]. A study reported that melatonin significantly improved total dry matter accumulation and wheat yield by 5.9 and 14.9% respectively in Yangmai-18, whereas by 3.2 and 26.0%, respectively in Yannong − 19 [34]. The response of melatonin on drought-resistant seedlings varies with the drought sensitivity of wheat cultivars, involving complex physiological mechanisms to maintain water status [31]. Melatonin application modulates growth and development and enhances resistance to abiotic stresses in harsh environments [31]. The necessity to overcome these stresses has generated considerable scientific interest in further exploring melatonin’s role in enhancing plant stress resistance [32]. Despite extensive research into melatonin’s effects on plant physiology and various growth and stress response compounds, a significant gap persists in our understanding of its impact on photosynthetic parameters.

However, its effects on wheat- a crop of globally significance has yet to be comprehensively synthesized or analyzed through meta-analysis. This study aims to elucidate the effects of exogenously applied melatonin on various physiological aspects of wheat. These factors were evaluated under different environmental conditions, including different day and night temperatures, application methods, concentration of melatonin, stress types, humidity, and wheat varieties. The primary goal is to comprehensively analyze the effect of melatonin on key physiological parameters of wheat crop. This includes identifying application methods that optimize antioxidant enzymes and improve photosynthetic parameters, chlorophyll fluorescence, leaf water status, and photosynthetic pigments in wheat. Additionally, this study seeks to determine the optimal concentration of melatonin that maximizes enzymatic activity, decreased ROS, and improved photosynthetic performance.

## Materials and methods

### Database construction and literature search

In this meta-analysis, we concentrated on evaluating the impact of melatonin on various aspects of wheat physiology, including photosynthetic parameters (photosynthetic rate, stomatal conductance, transpiration rate, and intercellular CO_2_ concentration), chlorophyll fluorescence (non-photochemical quenching, photochemical quenching, fluorescence yield, and quantum yield of PDII), leaf water status (leaf relative water content, water potential, osmotic potential, and water use efficiency), and photosynthetic pigments (total chlorophyll, chlorophyll a, chlorophyll b, carotenoid, and chlorophyll SPAD). The goal was to quantitatively determine the overall effect size of melatonin on these parameters. This involved evaluating variations based on experimental conditions i.e. controlled condition (greenhouse/incubator with control temperature and humidity) and natural condition (greenhouse/field study with natural temperature and humidity), melatonin concentrations, application methods, stress types, day and night temperature, humidity, and wheat varieties. We sought to identify trends and patterns, such as correlation between the effect size (ln*RR*) of photosynthetic rate and various chlorophyll pigments (total chlorophyll, chlorophyll a, chlorophyll b, and carotenoid), stomatal conductance, transpiration rate, intercellular CO_2_ concentration, and water use efficiency. This study also aimed to elucidate the relationship between melatonin concentration and photosynthetic rate, chlorophyll pigments, stomatal conductance, transpiration rate, intercellular CO_2_ concentration, and water use efficiency under varying environmental stress conditions. Based on the findings, provide practical recommendations for using melatonin to enhance wheat resilience and improve photosynthetic parameters, chlorophyll fluorescence, leaf water status, and photosynthetic pigments.

A systematic literature search was conducted across several databases including Web of Science, PubMed, Cochrane Library, and Scopus, targeting publications up to November 2023. Search terms included melatonin, plant photosynthetic rate, stomatal conductance, transpiration rate, fluorescence yield, leaf relative water content, water potential, osmotic potential, water use efficiency, total chlorophyll, chlorophyll a, chlorophyll b, carotenoid, chlorophyll SPAD, intercellular CO_2_ concentration, and wheat. The initial search identified 177 studies. After applying inclusion and exclusion criteria that prioritized studies with control groups (no melatonin) and specified parameters related to wheat’s physiological responses to melatonin, we selected 31 relevant studies. Studies lacking clear control or fewer than three replications were excluded. Review, non-experimental studies, articles not in English, and those lacking essential data for calculating effect size were also excluded. Additional variables were collected from the studies including melatonin concentration (µM), experimental sites, stress types (e.g. drought, salinity, heavy metal, cold and heat temperature, waterlogging, and infrared radiation stress), and environmental conditions such as humidity and day/night temperatures.

### Data extraction and statistical analysis

The selected studies were scrutinized, and data relating to sample size (n), means, standard deviation (SD), and/or standard error (SE) for each parameter including photosynthetic parameters, chlorophyll fluorescence, leaf water status, and photosynthetic pigments were extracted from both treatment and control groups. When the data were presented in figure form the GetData graph digitizer software version 2.26 was used to accurately extract the mean and SD or SE data. The following formula was used to convert SE to SD for both control and treatment groups when only SE was reported.

$$SD = SE \times \surd n$$

Effect sizes were computed using the log response ratio (ln*RR*):

$$\text{l}\text{n}RR=ln\left(\frac{{Mean}_{m}}{{Mean}_{c}}\right)$$

In this context ‘n’ represents the number of replications, ‘m’ denotes the treatment group, and ‘c’ indicate the control group. The variance of log response ratio (Vln*RR*) was calculated using the following equation [61, 62]:

$$\text{V}\text{l}\text{n}RR=ln\left(\frac{{SD}_{m}^{2}}{{n}_{m.}{Mean}_{m}^{2}}+\frac{{SD}_{c}^{2}}{{n}_{c}{. Mean}_{c}^{2}}\right)$$

Weights (W) for each study were the inverse of Vln*RR* and was calculated using the equation [63, 64]:

$$\text{W}= \frac{1}{\text{V}\text{l}\text{n}RR}$$

The overall mean effect size (ln*RR*_++_), was calculated using a random-effects model, which incorporates both within-study and between-study variability [61]:

$${\text{l}\text{n}RR}_{++}=\frac{\sum (W.\text{l}\text{n}RR)}{\sum W}$$

The standard deviation of the overall log response ratio, denoted as SDln*RR*_++_, was estimated by taking the square root of the inverse of the sum of the weights:

$${SD}_{{lnRR}_{++}}=\sqrt{\frac{1}{\sum \text{W}}}$$

Heterogeneity across studies was quantified using the Q statistic [61], which follows a chi-square distribution with k^− 1^ degrees of freedom, where k is the number of studies:

$$Q = \sum (W \cdot {({\rm{ln}}RR - {\rm{ln}}R{R_{ + + }})^2})$$

We interpreted heterogeneity using the I² statistic, which describes the percentage of total variation across studies due to heterogeneity rather than chance:

$${I}^{2} =\frac{Q-\left({k}^{-1}\right)}{Q}\times 100\%$$

We conducted subgroup analyses and meta regressions to explore potential sources of heterogeneity. Publication bias was evaluated with funnel plots and Egger’s regression test. All statistical analyses were performed using R software, specifically ‘metafor’ package, with a significance level set at *p* < 0.05. Results are presented with 95% bootstrapped confidence intervals (CIs). For graphical representation and analysis of the effect of melatonin on photosynthetic parameters, chlorophyll fluorescence, leaf water status, and photosynthetic pigments in wheat, we utilized the ‘ggplot2’, ‘tidyr’, and ‘dplyr’ packages in R, which are renowned for their capability to produce complex and informative graphics.

## Results

### Data heterogeneity and publication bias

Our meta-analysis revealed significant heterogeneity across the studies in several categories including overall effect, photosynthetic parameters, chlorophyll fluorescence, leaf water status, and photosynthetic pigments (Table 1). For the overall effects, the I² value reached 99.90%, with a tau² of 0.07 (± 0.0044), signifying a high degree of variability that extends beyond random variation. Specifically, for photosynthetic parameters, the I² was 98.56% with a tau² of 0.05 (± 0.0096); for chlorophyll fluorescence, I² was 99.93% with a tau² of 0.02 (± 0.0029); for leaf water status, I² reached 99.77% with a tau² of 0.12 (± 0.0223); and for the chlorophyll pigments, I² was 99.02% with a tau² of 0.05 (± 0.0049). These high I² values, alongside significant Q statistics (*p* < 0.0001), underline the substantial heterogeneity across the categories, justifying the use of random-effects models in our analysis. Furthermore, we calculated subgroup heterogeneity for factors such as melatonin concentration, application method, experimental conditions, stress types, day and night temperature, humidity of experimental site, and verity, for photosynthetic parameters, chlorophyll fluorescence, leaf water status, and photosynthetic pigments (Table S1).

**Table 1:** Quantitative synthesis of publication bias and heterogeneity analysis of overall, chlorophyll content, hydration dynamics, photosynthetic parameters, photosynthetic light quenching mechanism, and gas exchange parameters in wheat

Variables	Q	df	tau^2^	I^2^ (%)	H^2^	*p*-value
Overall	23878.37	548	0.0 7 ± 0.0044	99.90	1039.85	< 0.0001
Photosynthetic parameters	5018.08	170	0.05 ± 0.0096	98.56	69.33	< 0.0001
Chlorophyll fluorescence	723.49	72	0.02 ± 0.0029	99.93	1493.24	< 0.0001
Leaf water status	3043.31	64	0.12 ± 0.0223	99.77	435.78	< 0.0001
Photosynthetic pigments	10783.68	239	0.05 ± 0.0049	99.02	102.55	< 0.0001

Note: Total heterogeneity (Q), degree of freedom (df), estimated amount of total heterogeneity (tau^2^), total heterogeneity / total variability(I^2^), total variability / sampling variability (H^2^), and *p*-value

The funnel plot exhibited asymmetry (Figure S1), which was statistically confirmed by Egger’s regression test (Table 2). For the overall effect, significant asymmetry was observed (t = 11.31, df = 547), with a small intercept (b = 0.0008) and a confidence interval (CI) ranging from −0.0034 to 0.0049, suggesting notable bias as shown by a highly significant *p*-value (< 0.0001). For photosynthetic parameters, significant asymmetry was also detected (t = 5.51, df = 169) with a larger intercept (b = -0.0077), suggesting potential publication bias given the highly significant *p*-value (< 0.0001). Chlorophyll fluorescence showed significant asymmetry as well (t = 3.02, df = 71) with a small intercept (b = 0.0029) and a highly significant *p*-value (0.0035) indicating that smaller studies may disproportionately influence the overall effect size. Similarly, leaf water status (t = 2.38, df = 63, *p* = 0.0286) and chlorophyll pigments (t = 5.91, df = 238, *p* < 0.0001) showed a significant asymmetry, indicating apparent publication bias (Table 2).

**Table 2:** Regression analysis for funnel plot asymmetry of overall, chlorophyll content, hydration dynamics, photosynthetic parameters, photosynthetic light quenching mechanism, and gas exchange parameters in wheat

Variables	t	df	b	CI	*p*-value
Overall	11.31	547	0.0008	-0.0034 to 0.0049	< 0.0001
Photosynthetic parameters	5.51	169	-0.0077	-0.0472 to 0.0318	< 0.0001
Chlorophyll fluorescence	3.02	71	0.0029	0.0006 to 0.0052	0.0035
Leaf water status	2.38	63	-0.0639	-0.0992 to -0.0286	0.0286
Chlorophyll pigments	5.91	238	0.0395	0.0167 to 0.0623	< 0.0001

Note: Test for funnel plot asymmetry (t), degree of freedom (df), intercept from the regression line in Egger’s test (b), and t-statistic (t)

### Melatonin reverses the effect of abiotic stress on photosynthetic parameters

**Fig. 1:**
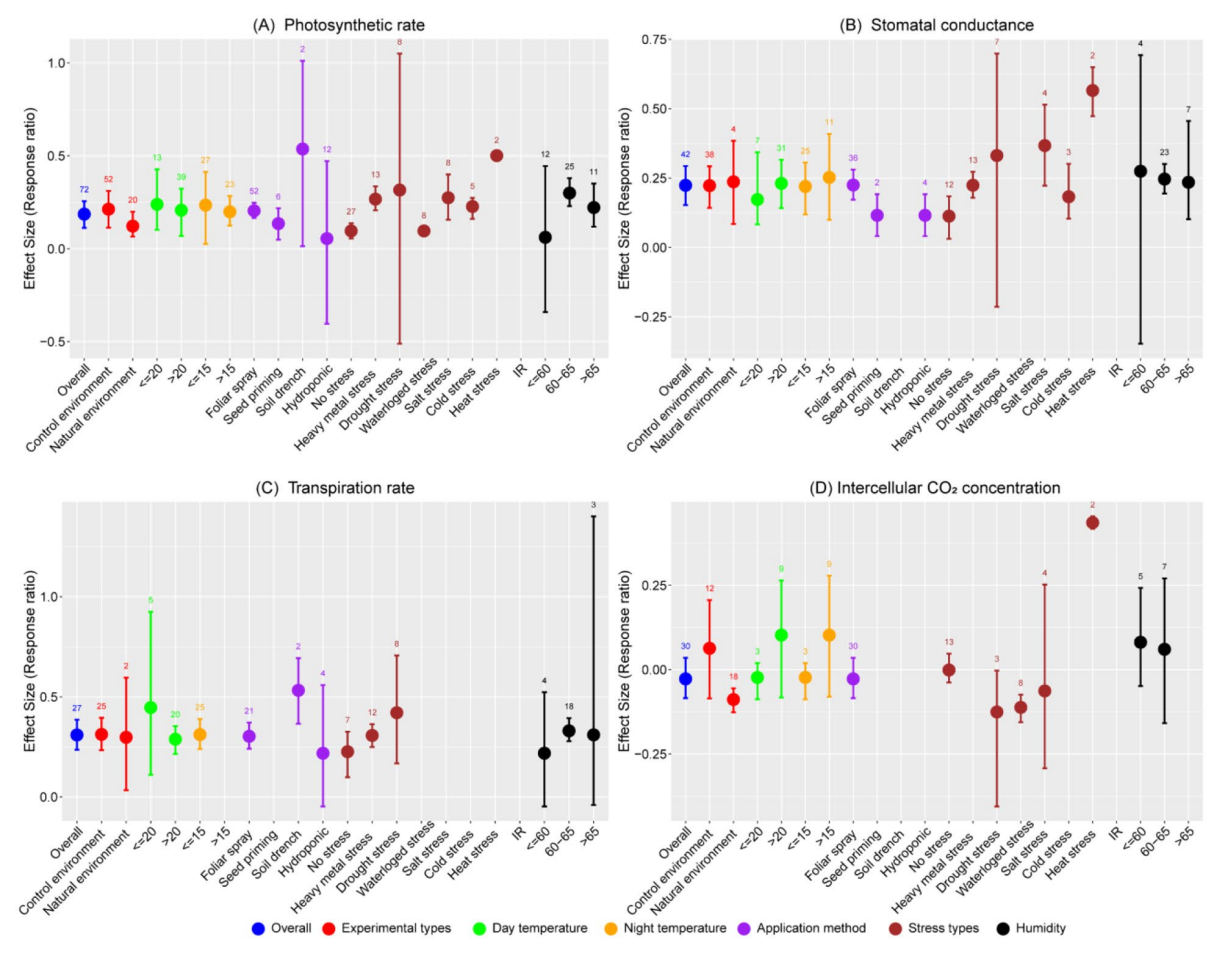
Response ratio of melatonin compared to control (no melatonin) with bootstrapped 95% confidence interval on photosynthetic rate (**A**), stomatal conductance (**B**), transpiration rate (**C**), and intercellular CO_2_ concentration (**D**) under different experimental conditions (Natural and control environment), temperatures (day and night temperature), melatonin application methods, stress types, and humidity of the experimental site. The zero (0) line (ln*RR*_++_ = 0) indicates no difference between melatonin and control. Numbers following the bar indicate the number of observations for each comparison

Melatonin application consistently enhances physiological parameters in wheat. Our findings reveal significant increases in the overall In*RR*_++_ of photosynthetic rate, stomatal conductance, and transpiration rate, with increases of 20.49, 25.09, and 36.29% respectively compared to the control (Fig. 1A-C). Controlled environmental conditions consistently increased In*RR*_++_, ranging from 1.05 to 36.68%, with the most notable increase in transpiration rate of 36.68%. Under natural environmental conditions, enhancements in In*RR*_++_ varied from 12.96 to 34.70%, with transpiration rate again showing the highest increase. However, in controlled environments, melatonin had no significant effect on intercellular CO_2_ concentration, whereas in natural environments, it decreased by 8.47% compared to control (Fig. 1D). Day temperatures ≤ 20 °C significantly elevated In*RR*_++_ across all physiological parameters, with increases of 16.96% in photosynthetic rate and 68.37% in transpiration rate, while stomatal conductance decreased by 27.42% compared to temperatures > 20 °C. Similarly, night temperatures ≤ 15 °C led to increases of 20.31% in photosynthetic rate but resulted in a 14.42% decrease in stomatal conductance compared to warmer nights (temperatures > 15 °C). However, temperature and application method did not significantly affect intercellular CO_2_ concentrations.

Different melatonin application methods significantly influenced physiological parameters. For instance, foliar spray significantly enhanced photosynthetic rate and stomatal conductance by 56.29 and 105.52% respectively, compared to seed priming. Melatonin application with soil drenching increased photosynthetic rate by 213.37% over foliar spray and 389.77% over seed priming. However, hydroponic application did not significantly affect photosynthetic and/or transpiration rates relative to the control. Under no stress conditions, melatonin application led to increases in photosynthetic rate, stomatal conductance, and transpiration ranging from 10.09 to 25.36%, without significantly affecting intercellular CO_2_ concentration compared to the control. Melatonin application under heat stress induced significant increases in photosynthetic rate, stomatal conductance, and intercellular CO_2_ concentration by 65.9, 76.07, and 54.64% respectively, compared to control (Fig. 1A and B). Moreover, under drought stress, the transpiration rate increased by 52.30%, while the intercellular CO_2_ concentration decreased by 11.79% compared to the control (Fig. 1C and D). Similarly, waterlogging stress led to a 10.59% decrease in intercellular CO_2_ concentration relative to the control. These results suggest that melatonin significantly enhances wheat’s resilience to various abiotic stresses, particularly in moderate humid conditions (60–65%). Melatonin as a foliar spray proved especially effective method, markedly increasing photosynthetic rate, stomatal conductance, and transpiration rate, while soil drenching yielded the most significant increases in photosynthetic and transpiration rates. These results indicate that melatonin could be a valuable growth regulator in improving wheat’s resilience to environmental stressors across diverse wheat varieties (Table S2). Melatonin application significantly enhanced photosynthetic parameters across all wheat varieties, with the exception of Yan 995, Aikang58, Sids 14, Yangmai 18, and Yannong 19, which showed no significant effect on intercellular CO_2_ concentration. Additionally, Hengguan35 and Yan 995 displayed no significant impact on photosynthetic rate; Jimai 22 exhibited no change in stomatal conductance; and both Hengguan35 and Yan 995 showed no alteration in transpiration rate compared to control (Table S2).

### Regulatory effect of melatonin on chlorophyll fluorescence

**Fig. 2:**
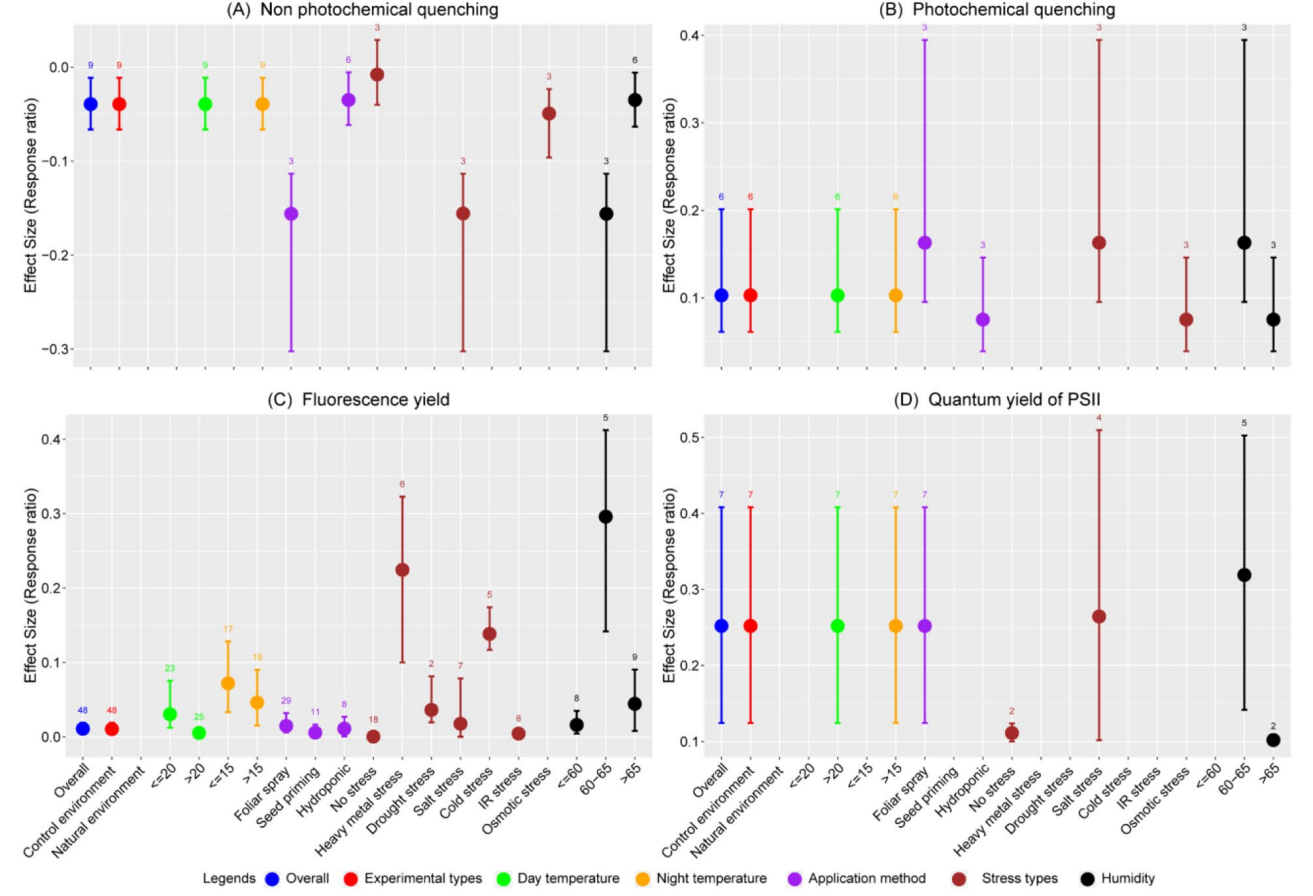
Response ratio of melatonin compared to control (no melatonin) with bootstrapped 95% confidence interval on non-photochemical quenching (**A**), photochemical quenching (**B**), fluorescence yield (**C**), and quantum yield of PSII (**D**) under different experimental conditions (Natural and control environment), temperatures (day and night temperature), melatonin application methods, stress types, and humidity of the experimental site. The zero (0) line (ln*RR*_++_ = 0) indicates no difference between melatonin and control. Numbers following the bar indicate the number of observations for each comparison

Melatonin treatment has variably yet significantly influenced chlorophyll fluorescence parameters. Overall, non-photochemical quenching (In*RR*_++_) displayed a slight decrease of 3.85%, whereas photochemical quenching, fluorescence yield, and quantum yield of PSII experienced increases of 10.84%, 0.11%, and 28.66%, respectively, in comparison to the control (Fig. 2A-D). Excluding no-stress conditions, all other parameters led to a notable reduction in non-photochemical quenching. Furthermore, variations in day and night temperatures notably influenced fluorescence parameters; both lower and higher temperatures resulted in an increase in photochemical quenching, fluorescence yield, and quantum yield, while decreased non-photochemical quenching.

Regarding application methods, all approaches except seed priming, which showed no significant difference, resulted in increased photochemical quenching and quantum yield. Under no-stress conditions, melatonin application substantially enhanced the quantum yield of PSII by 11.76%. Under salt stress, non-photochemical quenching decreased by 14.4%, while photochemical quenching, fluorescence yield, and quantum yield of PSII were increased by 17.7%, 1.76%, and 30.26%, respectively. Osmotic stress also led to a noteworthy 4.81% decrease in non-photochemical quenching and 7.8% increase in photochemical quenching (Fig. 2A, B). Lastly, humidity levels significantly influenced the fluorescence parameters; both moderate (60–65%) and high (> 65%) humidity conditions enhanced photochemical quenching, fluorescence yield, and quantum yield of PSII, with moderate humidity demonstrating a more significant impact (Fig. 2A-D). The application of melatonin enhanced the fluorescence yield in wheat varieties ANK-32B, Bezostaja-1, Pandas, Sids_14WH_542, Xinong 9871, and Yan 995 compared to the control (Table S3). Conversely, the varieties Chuannong_19 and Sids_14 experienced a decrease in non-photochemical quenching and a significant increase in photochemical quenching.

### Ameliorative effect of melatonin on leaf water status

**Fig. 3:**
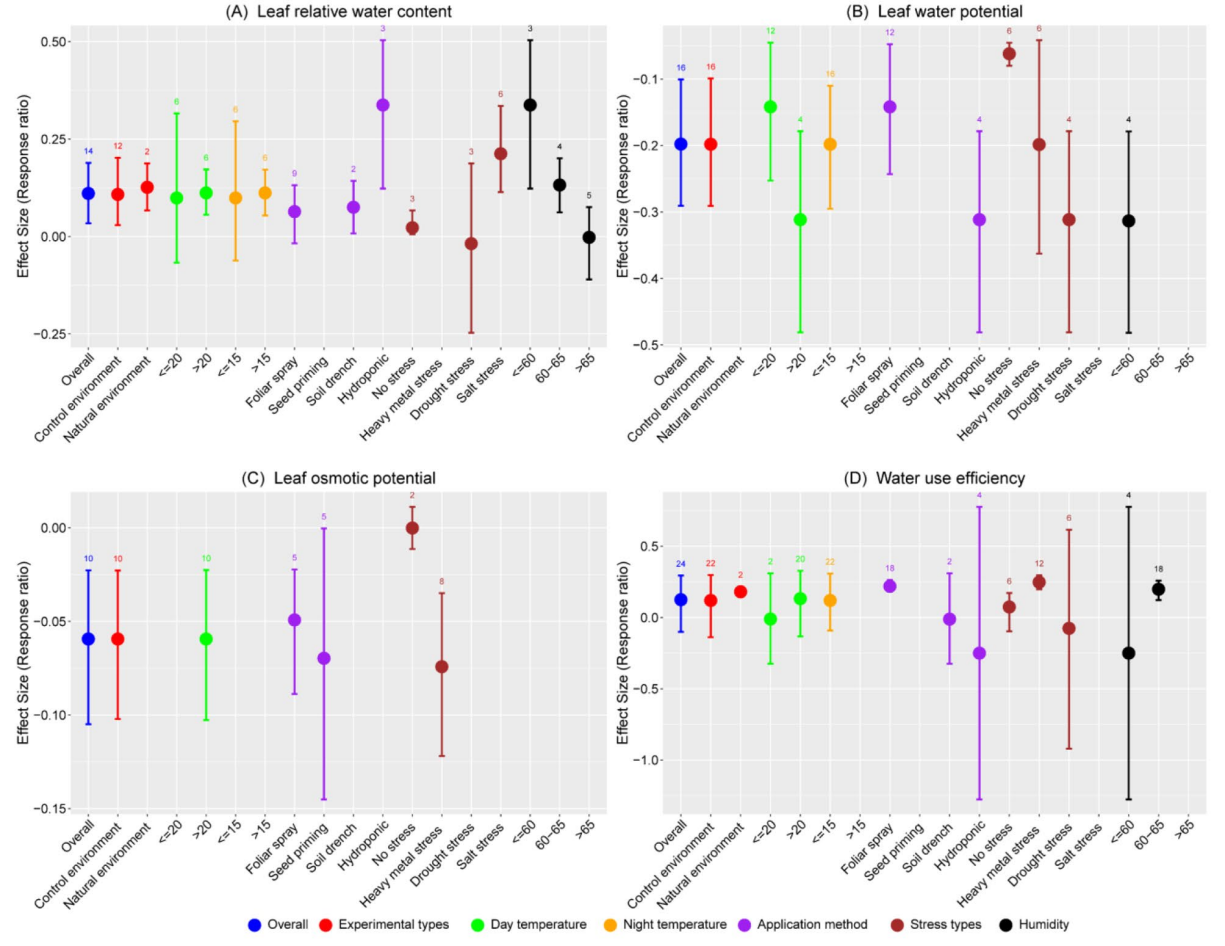
Response ratio of melatonin compared to control (no melatonin) with bootstrapped 95% confidence interval on leaf relative water content (**A**), leaf water potential (**B**), leaf osmotic potential (**C**), and water use efficiency (**D**) under different experimental conditions (Natural and control environment), temperatures (day and night temperature), melatonin application methods, stress types, and humidity of the experimental site. The zero (0) line (ln*RR*_++_ = 0) indicates no difference between melatonin and control. Numbers following the bar indicate the number of observations for each comparison

The overall In*RR*_++_ for leaf relative water content showed significant increase of 11.68% compared to control (Fig. 3A). This effect was more pronounced under hydroponic conditions, showing a substantial 40.13% increase, suggesting that hydroponic system may enhance water retention in leaves under abiotic stress. Melatonin significantly increased leaf relative water content in both experimental conditions compared to control, yet no significant difference was observed between control and natural environments. Melatonin’s impact varied across temperature ranges; temperature above 20 °C during the day and above 15 °C at night displayed an 11.84% increase over control, while lower temperatures showed no significant effect. Overall, a significant decrease in leaf water potential was noted (In*RR*_++_ = -0.1977), reflecting a reduction of 17.94% from the control (Fig. 3B). Extreme temperatures above 20 °C, were associated with a more pronounced decrease in leaf water potential (In*RR*_++_ = -0.3114), representing a 26.76% decline. These results suggest that higher temperatures may exacerbate leaf water stress in wheat. The overall effect on leaf osmotic potential was negative (In*RR*_++_ = -0.0594), indicating a 5.77% reduction from the control, indicating that melatonin tends to lower osmotic potential in wheat leaves. Melatonin application significantly decreased the leaf osmotic potential across all application methods under abiotic stress (Fig. 3C). Regarding water use efficiency, melatonin application did not significantly impact overall In*RR*_++_, as the lower limit crosses the zero line. However, a remarkable increase was observed in natural environmental conditions (In*RR*_++_ = 0.1806), resulting in a 19.79% increase over control (Fig. 3D). These results suggest that melatonin application facilitate more efficient water use in natural versus controlled environmental conditions. In addition, moderate humidity (60–65%) was associated with improved water use efficiency (In*RR*_++_ of 0.1983), corresponding to a 21.93% increase over control, underscoring the role of ambient humidity in water use efficiency.

In hydroponic system, melatonin application was the most effective, increasing leaf relative water content by 40.13% and decreasing water potential by 26.76% compared to control. This effect was contrastingly higher than other application methods. Seed priming with melatonin resulted in much higher reduction (6.73%) in leaf water potential compared to foliar application (4.8%). Different stress types have variable effects on different parameters. The meta-analysis showed that melatonin application significantly increased leaf relative water content by 23.66% under salt stress and improved water use efficiency by 28.11% under heavy metal stress. In contrast, it decreased leaf water potential by 17.99 and 26.76% in heavy metal and drought stress, respectively, and reduced leaf osmotic potential by 7.15% under heavy metal stress. These results suggest a protective effect of melatonin in mitigating the adverse effects of abiotic stress in wheat. Moreover, lower humidity significantly increases leaf relative water content by 40.13% and decreases leaf water potential by 26.70% with melatonin application (Fig. 3A and B).

### Exogenous melatonin enhances photosynthetic pigments

**Fig. 4:**
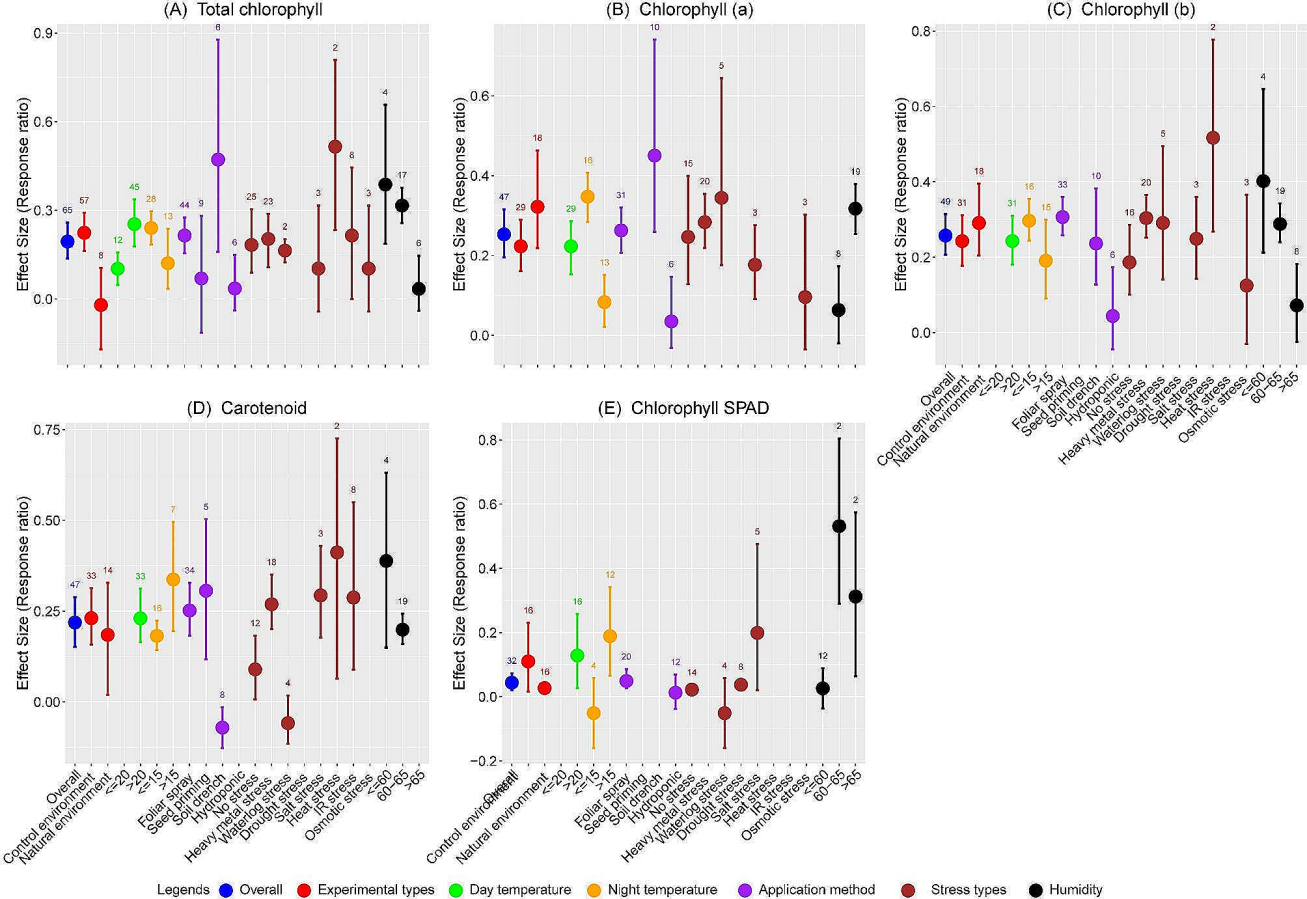
Response ratio of melatonin compared to control (no melatonin) with bootstrapped 95% confidence interval on total chlorophyll (**A**), chlorophyll a (**B**), chlorophyll b (**C**), carotenoid (**D**), and chlorophyll SPAD (**E**) under different experimental conditions (Natural and control environments), temperatures (day and night temperature), melatonin application methods, stress types, and humidity conditions at the experimental site. The zero (0) line (ln*RR*_++_ = 0) indicates no difference between melatonin and control. Numbers following the bar indicate the number of observations for each comparison

Our meta-analysis showed that melatonin application was associated with significant enhancement in overall pigment concentrations. Melatonin application significantly increased total chlorophyll content, chlorophyll a, and chlorophyll b by 21.47, 28.83, and 29.36%, respectively (Fig. 4). Carotenoids also showed a 24.46% increase compared to control. The impact of melatonin on total chlorophyll content was particularly notable in a controlled environment, with no significant changes observed under natural condition (Fig. 4A). However, melatonin significantly increased chlorophyll a, chlorophyll b, and carotenoid under both controlled and natural conditions compared to control (Fig. 4B and C). Temperature was a critical factor, with day temperatures over 20 °C leading to increases of 28.85% in total chlorophyll, 25.01% in chlorophyll a, 27.49% in chlorophyll b, and 25.89% in carotenoids. Melatonin application also increased the chlorophyll pigment with both low and high night temperatures, with temperatures ≤ 15 °C being particularly beneficial, with total chlorophyll, chlorophyll a, and chlorophyll b content increased by 27.18, 41.55 and 34.54%, respectively.

Soil drenching of melatonin exhibited the most pronounced effects, significantly boosting total chlorophyll and chlorophyll a by 60.30 and 56.91% increase over control, respectively. Compared to other method, foliar spray was also effective, particularly for chlorophyll b was increased by 35.89%, while seed priming was most effective for rising carotenoid content by 35.83%. Under no stress conditions, all types of chlorophyll pigments significantly increased, with chlorophyll a showing a 27.97% increase. Heavy metal stress led to a 32.80% increase in chlorophyll a (Fig. 4B). Melatonin application under heat stress had the most substantial impact, elevating total chlorophyll, chlorophyll b, and carotenoid by 67.45, 67.68, and 50.94%, respectively, compared to the control (Fig. 4A, C and D). Humidity showed substantial effects in response to melatonin with positive effects on pigment contents under low to moderate humidity levels (≤ 60% and 60–65%). Total chlorophyll, chlorophyll b, and carotenoids increased by 47.31 and 37.19%, 49.50 and 33.39%, and 47.46 and 22.01%, respectively, under these humidity conditions.

Melatonin application led to a moderate, yet significant increase of 4.49% in chlorophyll SPAD compared with control. In controlled environments, melatonin had a more substantial effect on chlorophyll SPAD (In*RR*_++_ = 0.1100), while natural environment exhibited a slight increase (In*RR*_++_ = 0. 0270). The influence of temperature on melatonin’s effectiveness was notable; higher day temperature (> 20 °C) facilitated a 13.71% improvement in chlorophyll SPAD (Fig. 4E). Similarly, under warmer night conditions (> 15 °C), a notable increase in chlorophyll SPAD was observed (In*RR*_++_ = 0.1887, 20.77% increase), suggesting a potential interaction between melatonin and temperature. Regarding application methods, foliar application yielded a modest increase in chlorophyll SPAD (In*RR*_++_: 0.0491, 5.03% increase over control), while hydroponic applications showed no significant changes.

In our meta-analysis of stress types, salt stress responded well to melatonin, exhibiting a 22.01% increase in chlorophyll SPAD, whereas drought stress showed no significant effect compared to control. Interestingly, high (> 65%) and moderate (60–65%) humidity conditions significantly boosted chlorophyll SPAD by 36.64 and 70.09%, respectively over control, suggesting that ambient moisture levels might interact with melatonin’s physiological role. These findings suggest that melatonin significantly enhances plant chlorophyll pigment, particularly in controlled environment. This effectiveness varies with the application method, types of stress, and wheat varieties (Table S4), with soil drenching and foliar spray showing the most substantial improvements (Fig. 4).

### Relationship of photosynthetic rate and melatonin concentration with key physiological parameters

**Fig. 5:**
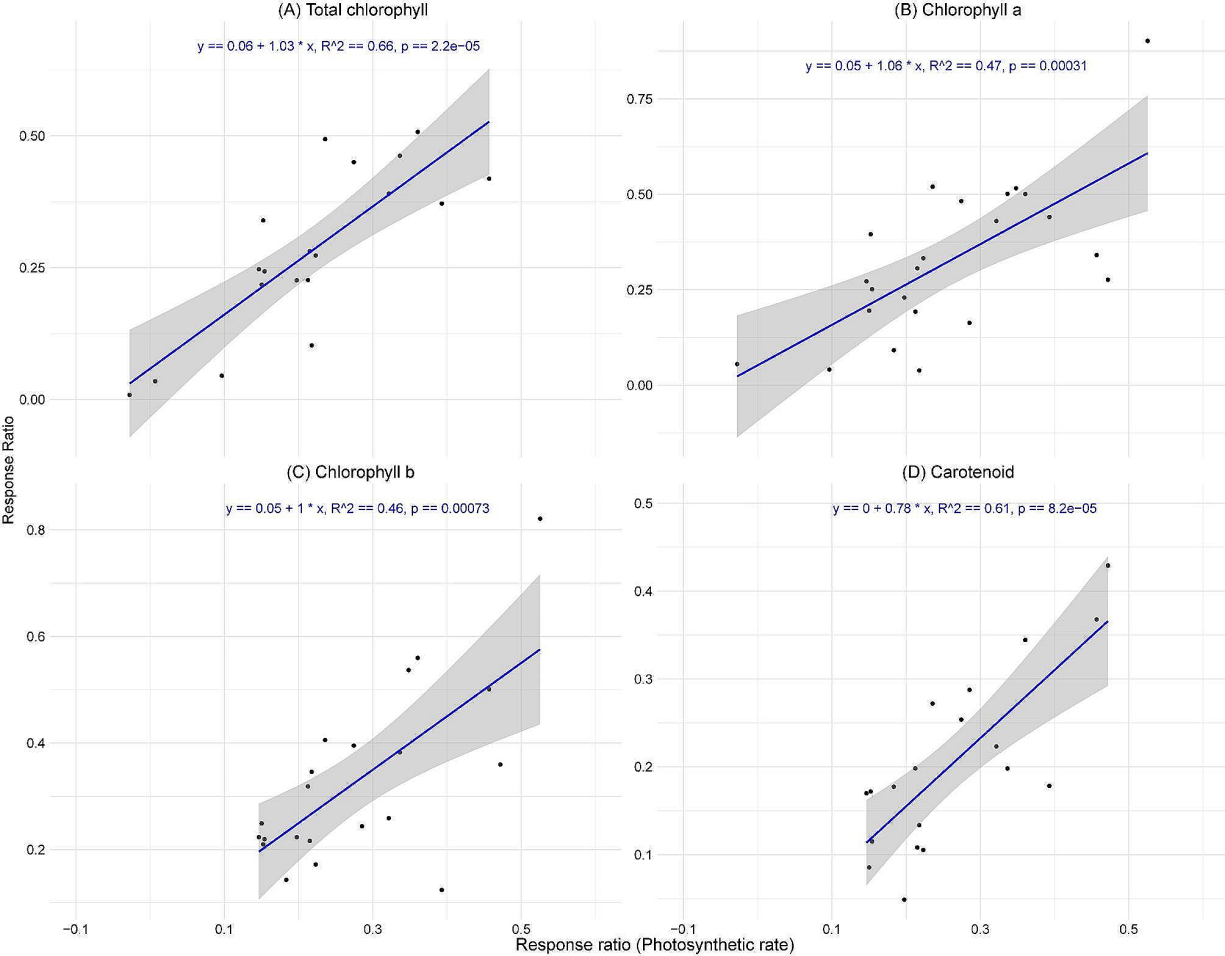
Relationship between the response rations of total chlorophyll (**A**), chlorophyll a (**B**), chlorophyll b (**C**), and carotenoid (**D**) with the response ratios of photosynthetic rate

The regression analysis showed a significant positive relationship between the photosynthetic rate and various chlorophyll pigments (Fig. 5). Total chlorophyll content was strongly correlated with photosynthetic rate (R^2^ = 0.66), suggesting that 66% of the variability in chlorophyll content was attributed to variations in the photosynthetic rate (Fig. 5A). These relationship predict a 1.03-unit increase in total chlorophyll for each unit increase in photosynthetic rate, with a highly significant *p*-value (*p* < 0.001). Similarly, chlorophyll a, chlorophyll b, and carotenoids also demonstrated significant positive correlations with photosynthetic rate (*p* < 0.001), with R^2^ value of 0.47, 0.046, and 0.61, respectively (Fig. 5B-D). These findings suggest that melatonin application enhances these key chlorophyll pigments, potentially boosting photosynthetic efficiency.

**Fig. 6:**
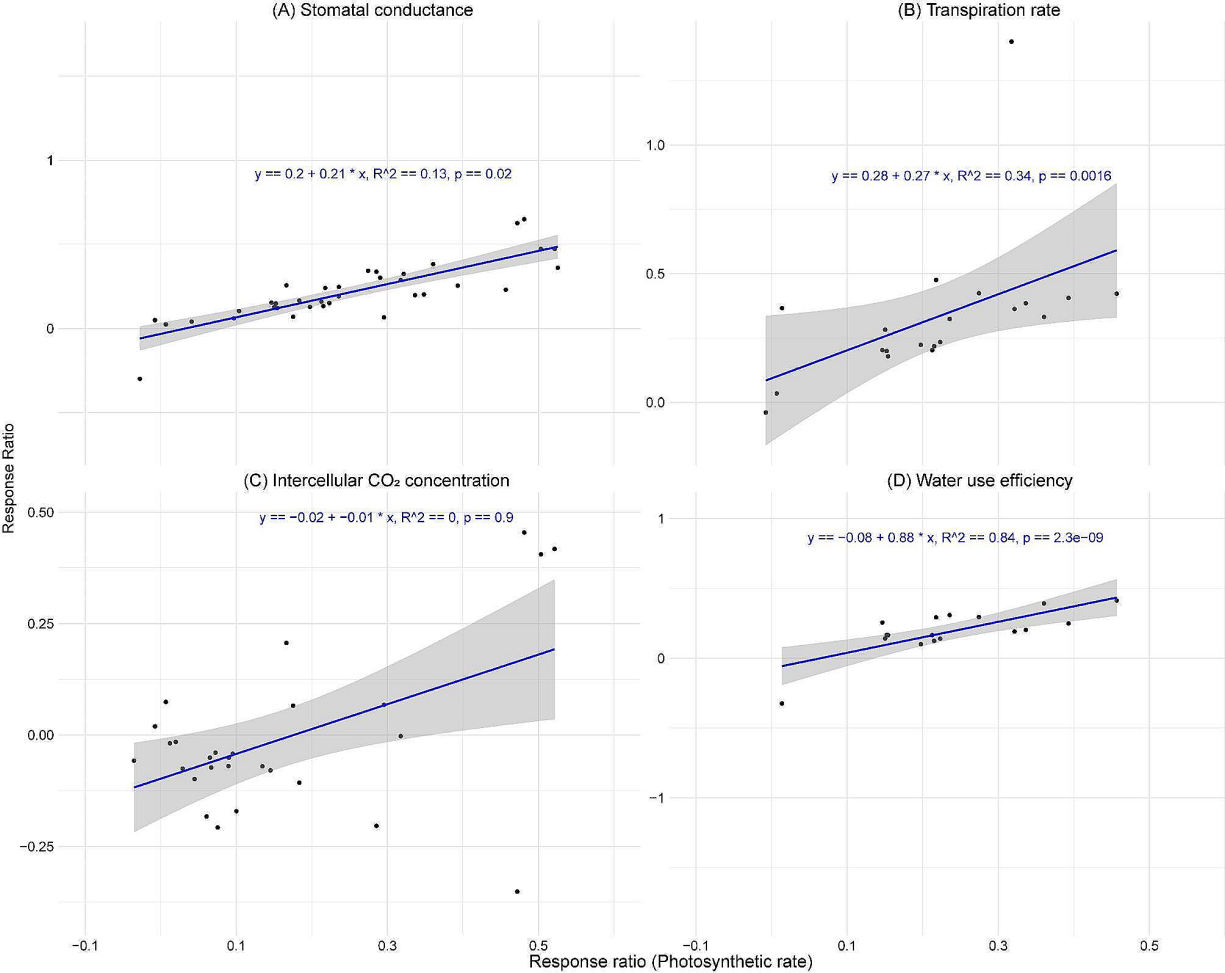
Relationship between the response rations of stomatal conductance (**A**), transpiration rate (**B**), intercellular CO_2_ concentration (**C**), and water use efficiency (**D**) with the response ratios of photosynthetic rate

The analysis of stomatal conductance showed a weak positive correlation with an R^2^ value of 0.13, indicating that only 13% of the variability in stomatal conductance was explained by photosynthetic rate (Fig. 6A). The transpiration rate exhibited a moderate positive relationship with a significant *p*-value (0.002) and an R^2^ value of 0.34, indicating that the photosynthetic rate explains 34% of variation in transpiration rate (Fig. 6B). However, the intercellular CO_2_ concentration did not exhibit a significant relationship with the photosynthetic rate (Fig. 6C). In contrast, water use efficiency displayed a strong positive relationship with photosynthetic rate (R^2^ value = 0.84, *p*-value < 0.001), demonstrating that the photosynthetic rate explains 84% of the variation in water use efficiency (Fig. 6D). In addition, regression analysis assessed the relationship between melatonin concentration and both the photosynthetic rate and chlorophyll pigments (Figure S2). This analysis showed that the photosynthetic rate, chlorophyll a, chlorophyll b, and carotenoids do not have significant relationship with melatonin concentration (Figures S2A, C-E). However, total chlorophyll content has a slightly higher R^2^ value of 0.06 and a marginal *p*-value of 0.046, suggesting a marginal statistical significance.

## Discussion

The meta-analysis revealed significant heterogeneity and signs of publication bias across studies examining the effects of melatonin on wheat physiology under various stress conditions. Elevated I^2^ values in parameters such as overall impact, photosynthetic parameters, chlorophyll fluorescence, leaf water status, and chlorophyll pigment suggest that melatonin’s impact is influenced by diverse factors, such as experimental conditions, application methods, stress types, melatonin concentrations, and genetic differences in wheat strains. These variations could be attributed to melatonin’s role in modulating photosynthetic efficiency and leaf water status through its involvement in antioxidant pathways and regulation of stomatal function [35–39]. Significant bias indicated by funnel plot asymmetry and Egger’s test, raises concerns about publication bias, echoing issues previously highlighted in agriculture research [40, 41]. This suggests that smaller studies may disproportionately affect overall effect size estimate. Melatonin seems to bolster the stress resilience of wheat, likely by enhancing physiological attributes such as the antioxidant defense system, which helps in combating oxidative stress. However, the specific mechanisms and the degree of melatonin’s influence under varied stress conditions remain unclear, highlighting a gap in our understanding of its role in plant physiology and underlying the need for more comprehensive research.

The meta-analysis confirms that melatonin significantly enhances wheat physiology under abiotic stress as indicated by improvements in photosynthetic rate, stomatal conductance, transpiration rate, and fluorescence yield. These findings align with those of I Ahmad, et al. [42] and ZA Buttar, et al. [36], who reported positive effects of melatonin on plant growth and chlorophyll contents under stress conditions. Melatonin likely boosts photosynthesis by increasing chlorophyll concentration and optimizing light absorption [37, 43]. Notable increases in transpiration rate in both controlled (36.68%) and natural (34.70%) environments reflect melatonin’s role in regulating water balance [16]. The varied response at different day and night temperatures suggests that melatonin might modulate temperature-responsive biochemical pathways, enhancing stress tolerance at higher temperatures [35, 44, 45]. The differential effectiveness of melatonin application methods, particularly the superiority of foliar spray over seed priming and the remarkable increase in transpiration rate with soil drenching, can be attributed to differences in melatonin absorption and translocation within the plant [40, 41, 46, 47]. The absence of significant effects on stomatal conductance under drought stress, contrasted with marked enhancements under heat stress, underscores the varied efficiency of melatonin under different stress conditions [11, 29, 48]. Overall, these results support melatonin’s potential as a growth regulator capable of enhancing wheat resilience to environmental stressors, corroborated by varying responses across different wheat varieties, which suggests the need for tailored melatonin application strategies [49, 50].

Melatonin significantly improves the leaf relative water content and modulates water potential in wheat, showing particularly strong effects under hydroponic and various abiotic stress conditions. This aligns with I Ahmad, et al. [42], who observed significant improvements in leaf water status under controlled environments. The substantial 40.13% increase in leaf water content under hydroponic conditions suggests a specialized role for melatonin in these systems, potentially due to its capacity to mitigate boron toxicity [16], cadmium and salt tolerance [51, 52]. Elevated temperatures (> 20 °C) with exacerbate water stress showed a decline of 26.76% in leaf water potential, echoing finding of ZA Buttar, et al. [36] that highlight the importance of environmental factors in melatonin’s function. Although overall water use efficiency was unaffected, but increases were observed under natural environmental conditions and moderate humidity, indicating melatonin’s potential to enhance water use efficiency in certain environments [53]. These results, supported by previous studies, demonstrate that zinc oxide nanoparticles combined with melatonin improve wheat growth under cadmium stress [54]. In addition, soil drenching and seed priming methods exhibit significant differences in their effects on water potential [41], and melatonin’s protective role under salt and heavy metal stress enhances water content and efficiency [11]. The observed variation in response to different stress types underline the complex interactions between melatonin and wheat physiology under stress.

Melatonin also significantly augments chlorophyll pigment concentration in wheat, as evidenced by notable increases in total chlorophyll, chlorophyll a, chlorophyll b, and carotenoid contents by 21.47, 28.83, 29.36, and 24.46%, respectively [16, 42, 48]. These enhancements are most pronounced under controlled environmental conditions, aligning with prior studies [35, 36, 55]. The impact of higher day temperature (> 20 °C) on chlorophyll content supports the observations by A Dradrach, et al. [11], suggesting temperature as a critical factor in melatonin’s efficacy. Notably, beneficial effects at night temperatures ≤ 15 °C on total chlorophyll and chlorophyll a content align with K Du, et al. [56], emphasizing the significance diurnal temperature variations. Soil drenching showed the highest increase in chlorophyll content in this meta-analysis, with results supported by CA Hansen, et al. [49] and N Iqbal, et al. [50], which highlight method-specific responses. Foliar spray and seed priming methos also demonstrated notable increases in different chlorophyll pigments, illustrating the diverse mechanisms of action [44, 57]. The increases in chlorophyll content under no stress and particularly under heat stress, with the highest enhancement in chlorophyll b, reflect melatonin’s protective role against abiotic stresses [40, 42, 56]. The variation in response to humidity levels further indicates the melatonin adaptability in different environmental conditions [58].

Additionally, our meta-analysis indicates that melatonin application has a nuanced effects on wheat physiology, enhancing chlorophyll SPAD and influencing intercellular CO_2_ concentration under various abiotic stress conditions. These findings align with I Ahmad, et al. [42], who reported the positive effects of melatonin on chlorophyll contents in wheat under salt stress. The 4.49% improvement in chlorophyll SPAD in controlled environments aligns with the findings of M Rizwan, et al. [59], emphasizing the influence of controlled conditions on melatonin’s efficacy. Higher day and night temperatures (> 20 °C and > 15 °C) enhance melatonin’s effectiveness, supporting observations by ZA Buttar, et al. [36] and F Chen, et al. [35] regarding the interplay of melatonin and environmental factors. In contrast, the decrease in intercellular CO_2_ concentration under drought and waterlogging stresses may reflect a stress-specific response [11, 60]. The differential efficacy of melatonin application methods, particularly the modest increase in chlorophyll SPAD with foliar application and no significant change in hydroponic conditions, highlights the importance of method selection [49]. Under heat stress, the significant increase in intercellular CO_2_ concentration could indicate an enhanced photosynthetic capability [50, 57]. Lastly, the significant enhancements in chlorophyll SPAD under high and moderate humidity further illustrate melatonin’s interaction with ambient moisture [44].

## Conclusions

This comprehensive meta-analysis elucidates melatonin’s complex role in augmenting wheat physiology under abiotic stress conditions. It explores how melatonin modulates photosynthetic parameters, chlorophyll fluorescence, leaf water status, and chlorophyll pigments, demonstrating its efficacy in improving plant resilience to stressful environments. Our analysis reveals significant heterogeneity and publication bias, highlighting the complex influence of experimental conditions, melatonin concentrations, and genetic variances in wheat varieties. Melatonin application enhances photosynthetic rates, stomatal conductance, and transpiration rates, with its effectiveness substantially influenced by environmental conditions, temperature ranges, and application methods. The study also shows that melatonin significantly ameliorated water stress by improving leaf water content and modulating water potential, thereby proving its utility in stress mitigation. Moreover, the pronounced increase in chlorophyll pigments emphasizes melatonin’s role in enhancing photosynthetic pigments and optimizing light absorption. The variability in response across different stress conditions and application methods suggests a need for tailored melatonin application strategies to fully leverage its potential as a growth regulator. Furthermore, the nuanced influence on chlorophyll SPAD and intercellular CO_2_ concentrations highlights the complex interaction between melatonin and plant physiological processes, justifying further investigation to elucidate the underlying mechanisms. Overall, this meta-analysis makes a significant contribution to our understanding of melatonin’s role in boosting the stress resilience of wheat crop. It sets the stage for future research aimed at optimizing melatonin application to promote sustainable agriculture practices.

### Electronic supplementary material

Below is the link to the electronic supplementary material.

Supplementary Material 1

## Data Availability

The entire dataset used and/or analyzed in the study is available from the corresponding author on reasonable request.
