# Supplementary material for: A meta-analysis of photosynthetic efficiency and stress mitigation by melatonin in enhancing wheat tolerance
Source: BMC Plant Biol. 2024 May 21;24:427. doi: 10.1186/s12870-024-05132-2 (PMC11106942; doi:10.1186/s12870-024-05132-2)
Supplement: Supplementary file 1 — Supplementary Material 1 [file 12870_2024_5132_MOESM1_ESM.docx]

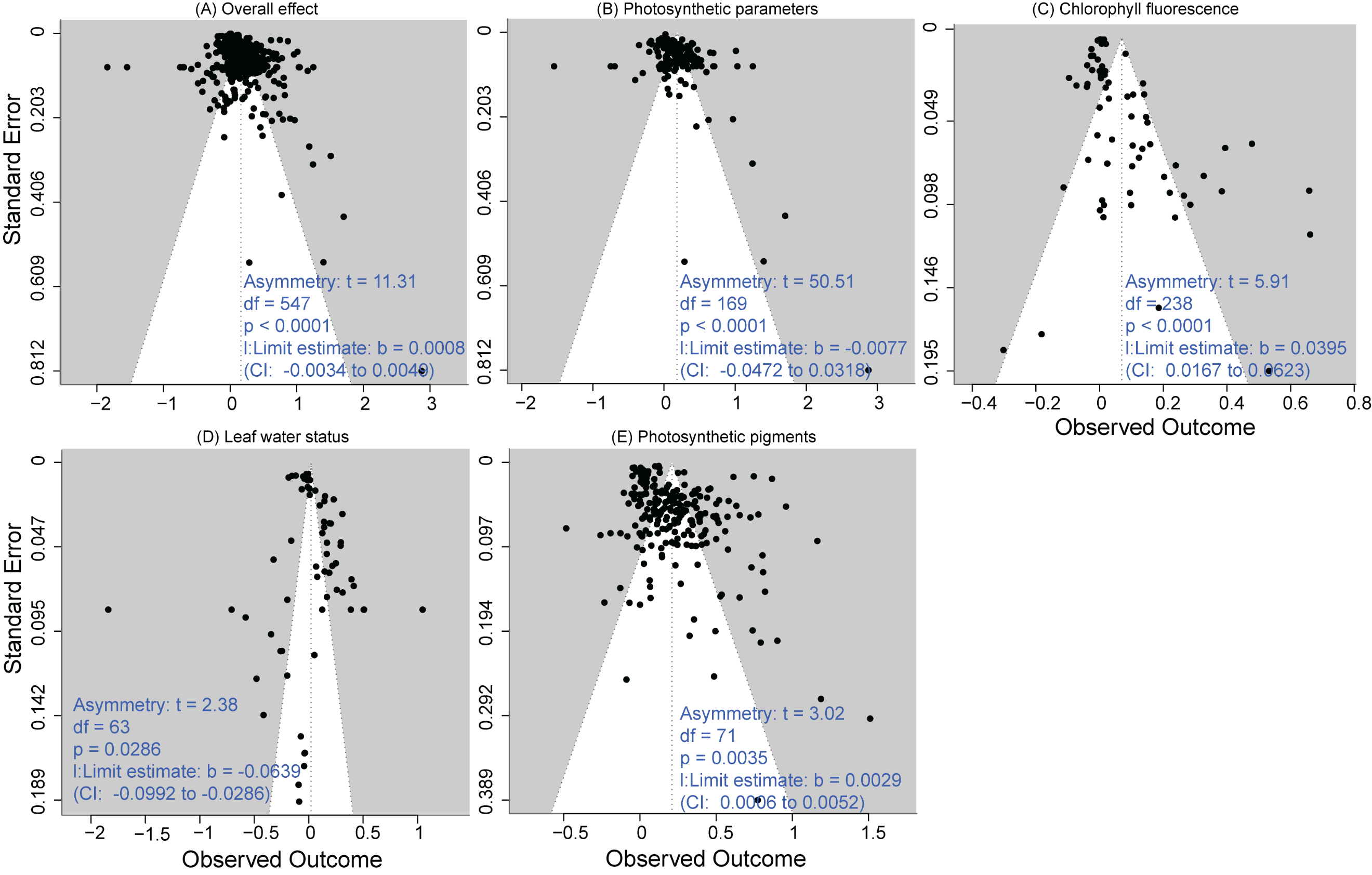


Figure S1. Illustration of funnel plot asymmetry due to heterogeneity, in the form of all overall effect (A), photosynthetic parameters (B), chlorophyll fluorescence (C), leaf water status (D), and photosynthetic pigments (E). Funnel plot including all studies (on the top) shows clear asymmetry (P < 0.001 for overall effect, photosynthetic parameters, and chlorophyll fluorescence while p > 0.05 for leaf water status and photosynthetic pigments from Egger test for funnel plot asymmetry).


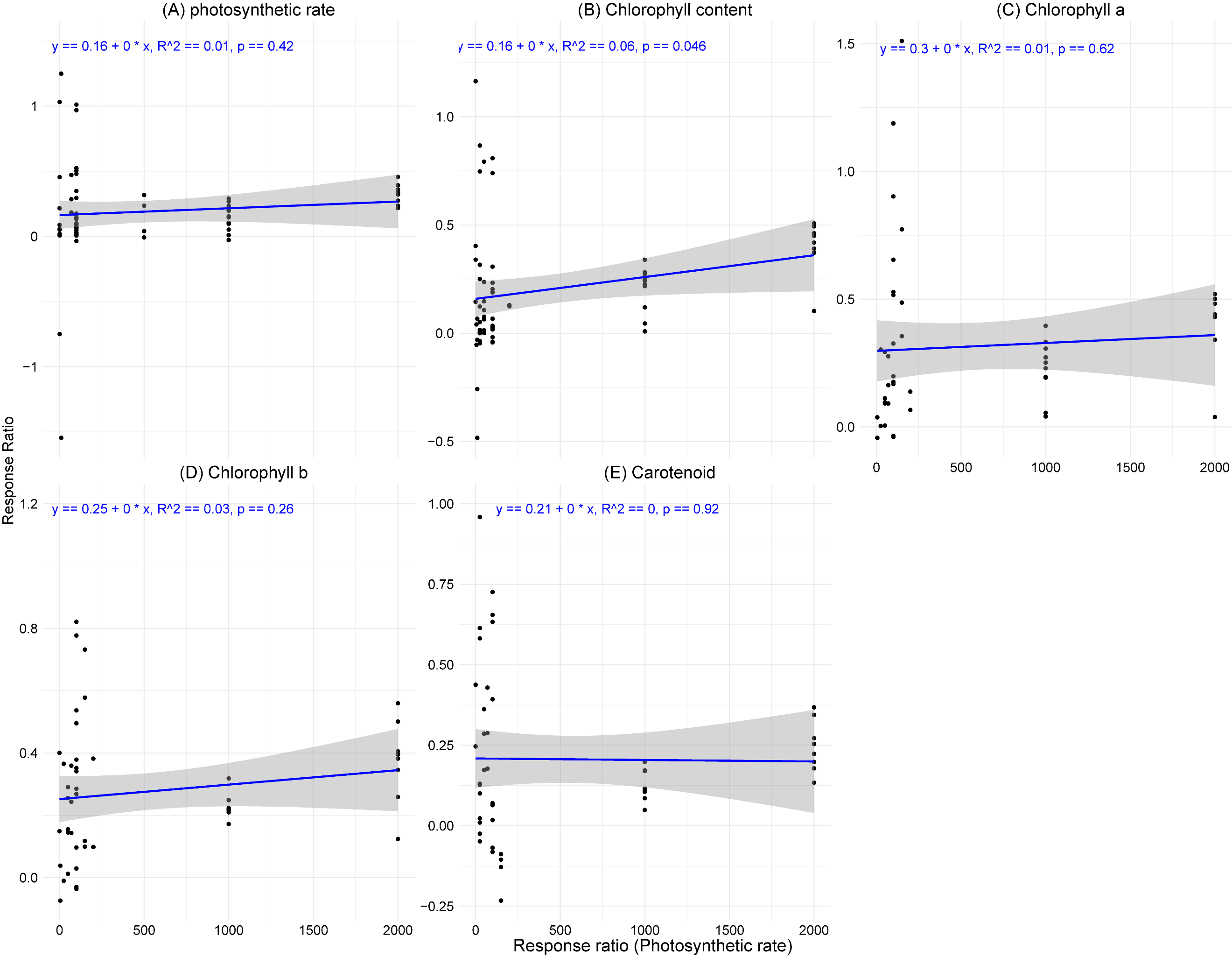


Figure S2. Relationship between the response rations of key photosynthetic rate (A), total chlorophyll (B), chlorophyll a (C), chlorophyll b (D), and carotenoid (E) with the response ratios of photosynthetic rate.

Table S1. Subgroup heterogeneity analysis of melatonin concentration, experimental conditions, melatonin application method, stress types, day and night temperatures, humidity, and varieties in wheat.

| Variables | QE | df | QM | df | p-value | tau^2^ | I^2^ (%) | H^2^ |
| --- | --- | --- | --- | --- | --- | --- | --- | --- |
| Overall | | | | | | | | |
| Melatonin concentration | 18817.69 | 535 | 292.04 | 14 | <0.0001 | 0.06 ± 0.0039 | 99.89 | 922.68 |
| Experimental conditions | 23810.13 | 547 | 200.34 | 2 | <0.0001 | 0.07 ± 0.0044 | 99.90 | 1035.69 |
| Application method | 21523.39 | 519 | 222.58 | 6 | <0.0001 | 0.07 ± 0.0044 | 99.89 | 948.48 |
| Stress types | 18405.64 | 535 | 323.53 | 14 | <0.0001 | 0.06 ± 0.0038 | 99.88 | 858.79 |
| Day temperature | 17772.67 | 417 | 222.41 | 10 | <0.0001 | 0.07 ± 0.0049 | 99.92 | 1273.38 |
| Night temperature | 6498.07 | 365 | 214.49 | 10 | <0.0001 | 0.06 ± 0.0050 | 98.66 | 74.45 |
| Humidity | 6506.3070 | 322 | 214.2803 | 7 | <0.0001 | 0.06 ± 0.0056 | 98.80 | 83.14 |
| Verity | 16414.98 | 516 | 500.81 | 33 | <0.0001 | 0.05 ± 0.0032 | 99.87 | 754.45 |
| Photosynthetic parameters | | | | | | | | |
| Melatonin concentration | 3825.97 | 164 | 75.23 | 7 | <0.0001 | 0.08 ± 0.0095 | 98.14 | 53.90 |
| Experimental conditions | 4586.60 | 169 | 78.51 | 2 | <0.0001 | 0.08 ± 0.0091 | 98.37 | 61.26 |
| Application method | 4529.74 | 166 | 70.87 | 5 | <0.0001 | 0.08 ± 0.0096 | 98.54 | 68.51 |
| Stress types | 3534.48 | 162 | 102.74 | 9 | <0.0001 | 0.07 ± 0.0087 | 98.25 | 57.10 |
| Day temperature | 3174.39 | 119 | 71.58 | 8 | <0.0001 | 0.10 ± 0.0132 | 97.84 | 46.38 |
| Night temperature | 2850.42 | 116 | 70.32 | 7 | <0.0001 | 0.0980 ± 0.0137 | 97.84 | 46.37 |
| Humidity | 2945.46 | 113 | 67.98 | 6 | <0.0001 | 0.10 ± 0.0141 | 97.95 | 48.78 |
| Verity | 2526.43 | 153 | 125.15 | 18 | <0.0001 | 0.07 ± 0.0083 | 97.75 | 44.41 |
| Chlorophyll fluorescence | | | | | | | | |
| Melatonin concentration | 609.11 | 65 | 41.01 | 8 | <0.0001 | 0.01 ± 0.0025 | 99.92 | 1296.61 |
| Experimental conditions | 4568.50 | 169 | 78.51 | 2 | <0.0001 | 0.08 ± 0.0091 | 98.37 | 61.26 |
| Application method | 694.17 | 70 | 34.35 | 3 | <0.0001 | 0.01 ± 0.0025 | 91.91 | 1110.96 |
| Stress types | 397.67 | 64 | 166.04 | 9 | <0.0001 | 0.01 ± 0.0005 | 99.52 | 208.08 |
| Day temperature | 577.38 | 63 | 32.99 | 10 | <0.0001 | 0.01 ± 0.0029 | 99.94 | 1582.115 |
| Night temperature | 303.33 | 53 | 55.44 | 8 | <0.0001 | 0.01 ± 0.0028 | 97.17 | 35.31 |
| Humidity | 242.47 | 42 | 52.32 | 5 | <0.0001 | 0.01 ± 0.0026 | 97.28 | 36.80 |
| Verity | 476.46 | 60 | 85.05 | 13 | <0.0001 | 0.01 ± 0.0015 | 99.87 | 775.54 |
| Leaf water status | | | | | | | | |
| Melatonin concentration | 1689.68 | 53 | 29.29 | 12 | <0.0001 | 0.09 ± 0.0191 | 99.70 | 337.94 |
| Experimental conditions | 3001.72 | 63 | 0.82 | 2 | <0.0001 | 0.12 ± 0.0226 | 99.78 | 445.13 |
| Application method | 2619.66 | 57 | 9.86 | 6 | <0.0001 | 0.12 ± 0.0227 | 99.73 | 371.30 |
| Stress types | 1917.41 | 58 | 13.77 | 7 | <0.0001 | 0.11 ± 0.0210 | 99.72 | 362.05 |
| Day temperature | 189932 | 55 | 16.30 | 6 | <0.0001 | 0.11 ± 0.0214 | 99.75 | 399.87 |
| Night temperature | 1361.53 | 46 | 2.51 | 5 | <0.0001 | 0.16 ± 0.0348 | 99.30 | 142.38 |
| Humidity | 1175.39 | 35 | 5.57 | 4 | <0.0001 | 0.18 ± 0.0429 | 99.52 | 209.86 |
| Verity | 1529.34 | 51 | 64.48 | 14 | <0.0001 | 0.06 ± 0.0138 | 99.59 | 244.05 |
| Photosynthetic Parameters | | | | | | | | |
| Melatonin concentration | 9076.43 | 228 | 280.29 | 12 | <0.0001 | 0.04 ± 0.0042 | 98.81 | 84.10 |
| Experimental conditions | 10380.34 | 238 | 199.91 | 2 | <0.0001 | 0.05 ± 0.0049 | 98.99 | 99.39 |
| Application method | 9141.19 | 213 | 200.68 | 5 | <0.0001 | 0.05 ± 0.0051 | 99.00 | 99.64 |
| Stress types | 8701.76 | 226 | 311.51 | 14 | <0.0001 | 0.04 ± 0.0040 | 98.71 | 77.29 |
| Day temperature | 8270.62 | 159 | 201.05 | 7 | <0.0001 | 0.04 ± 0.0051 | 98.90 | 90.77 |
| Night temperature | 1556.43 | 132 | 322.21 | 8 | <0.0001 | 0.02 ± 0.0030 | 92.80 | 13.88 |
| Humidity | 1277.07 | 117 | 292.22 | 7 | <0.0001 | 0.02 ± 0.0034 | 91.63 | 11.94 |
| Verity | 8268.11 | 216 | 384.71 | 24 | <0.0001 | 0.03 ± 0.0037 | 98.58 | 70.61 |

Note: Total heterogeneity (QE), test of moderators QM, degree of freedom (df), estimated amount of total heterogeneity (tau^2^), total heterogeneity / total variability (I^2^), total variability / sampling variability (H^2^), and p-value.

Table S2. Response ratio of melatonin compared to control with bootstrapped 95% confidence interval on photosynthetic parameters for different wheat varieties.

|  | Photosynthetic rate | | | Stomatal conductance | | | Transpiration rate | | | Intercellular CO_2_ concentration | | |
| --- | --- | --- | --- | --- | --- | --- | --- | --- | --- | --- | --- | --- |
| Varieties | N | RR | Bootstrap confidence intervals | N | RR | Bootstrap confidence intervals | N | RR | Bootstrap confidence intervals | N | RR | Bootstrap confidence intervals |
| Yan_995 |  |  |  |  |  |  |  |  |  | 3 | -0.0185 | -0.0185 to -0.0878 |
| Aikang58 | 2 | 0.2428 | 0.0068 to 0.9694 | 2 | 0.1736 | 0.0248 to 0.4199 | 2 | 0.2944 | 0.0341 to 0.596 | 2 | -0.2115 | -0.2115 to -0.4055 |
| ANK-32B | 2 | 0.1227 | 0.01 to 0.2357 |  |  |  |  |  |  |  |  |  |
| Bezostaja-1 |  |  |  |  |  |  |  |  |  |  |  |  |
| Giza 168 | 2 | 0.4224 | 0.3483 to 0.5254 | 2 | 0.278 | 0.2031 to 0.361 |  |  |  |  |  |  |
| GOAL | 2 | 0.3257 | 0.1752 to 0.5031 | 2 | 0.2646 | 0.0697 to 0.4736 |  |  |  | 2 | 0.235 | 0.235 to 0.0655 |
| Hengguan35 | 2 | -1.1479 | -1.5461 to -0.7496 | 2 | 0.6931 | 0.6931 to 0.6931 | 2 | 0.0967 | -0.069 to 0.2624 |  |  |  |
| Jimai 22 | 4 | 0.5766 | 0.8138 to 1.1116 | 2 | -0.1438 | -0.6931 to 0.4055 | 4 | 0.4423 | 0.0801 to 0.6991 |  |  |  |
| Jimai22 | 2 | 1.1402 | 1.0315 to 1.2489 |  |  |  |  |  |  |  |  |  |
| Lasani-2008 | 16 | 0.2525 | 0.2098 to 0.2989 | 16 | 0.209 | 0.1702 to 0.2489 | 16 | 0.3043 | 0.2583 to 0.3524 |  |  |  |
| Lianmai 6 | 4 | 0.108 | 0.0014 to 0.238 | 4 | 0.0378 | -0.1701 to 0.2255 |  |  |  |  |  |  |
| Pandas |  |  |  |  |  |  |  |  |  |  |  |  |
| Sids 14 | 3 | 0.3237 | 0.1835 to 0.4178 | 3 | 0.3145 | 0.1662 to 0.6262 |  |  |  | 3 | -0.2234 | -0.2234 to -0.3515 |
| WH 542 | 2 | 0.3256 | 0.1664 to 0.481 | 2 | 0.4493 | 0.2568 to 0.6495 |  |  |  | 2 | 0.3191 | 0.3191 to 0.2066 |
| WH-711 | 2 | 0.4084 | 0.2955 to 0.5213 | 2 | 0.2686 | 0.0664 to 0.4731 |  |  |  | 2 | 0.2433 | 0.2433 to 0.0677 |
| Wild type | 2 | 0.1537 | 0.0522 to 0.2683 |  |  |  |  |  |  |  |  |  |
| Xinong 9871 | 8 | 0.077 | 0.0329 to 0.1467 |  |  |  |  |  |  |  |  |  |
| Yan 995 | 3 | 0.1596 | -0.0076 to 2.875 | 3 | 0.2307 | 0.05 to 1.7047 | 3 | 0.2957 | -0.0397 to 1.4015 |  |  |  |
| Yangmai 18 | 8 | 0.0784 | 0.0481 to 0.1105 |  |  |  |  |  |  | 8 | -0.0495 | -0.0495 to -0.0638 |
| Yannong 19 | 8 | 0.0525 | 0.0214 to 0.0789 |  |  |  |  |  |  | 8 | -0.1171 | -0.1171 to -0.1604 |

The negative bootstrap values indicate no difference between melatonin and control. The ln*RR*_++_ is the response ratio, and N is the number of observations for comparison.

Table S3. Response ratio of melatonin compared to control with bootstrapped 95% confidence interval on fluorescence yield for different wheat varieties.

|  | Non photochemical quenching | | | Photochemical quenching | | | Fluorescence yield | | | Quantum yield of PSII | | |
| --- | --- | --- | --- | --- | --- | --- | --- | --- | --- | --- | --- | --- |
| Varieties | N | RR | Bootstrap confidence intervals | N | RR | Bootstrap confidence intervals | N | RR | Bootstrap confidence intervals | N | RR | Bootstrap confidence intervals |
| Yan_995 |  |  |  |  |  |  |  |  |  |  |  |  |
| Aikang58 |  |  |  |  |  |  |  |  |  |  |  |  |
| ANK-32B |  |  |  |  |  |  | 2 | 0.0284 | 0.0001 to 0.1054 |  |  |  |
| Bezostaja-1 |  |  |  |  |  |  | 10 | 0.0031 | 0.0001 to 0.0063 |  |  |  |
| Chuannong_19 | 6 | -0.0349 | -0.0634 to -0.0066 | 3 | 0.0752 | 0.039 to 0.1461 |  |  |  |  |  |  |
| Yangmai_18 |  |  |  |  |  |  |  |  |  |  |  |  |
| Yannong_19 |  |  |  |  |  |  |  |  |  |  |  |  |
| GOAL |  |  |  |  |  |  |  |  |  | 2 | 0.1019 | 0.1001 to 0.1038 |
| Lianmai 6 |  |  |  |  |  |  | 4 | 0.0709 | -0.0206 to 0.1536 |  |  |  |
| Pandas |  |  |  |  |  |  | 12 | 0.0887 | 0.0187 to 0.1721 |  |  |  |
| Sids_14 | 3 | -0.156 | -0.3023 to -0.1133 | 3 | 0.163 | 0.0953 to 0.3947 | 3 | 0.1825 | 0.1021 to 0.5322 | 3 | 0.3324 | 0.0991 to 0.6614 |
| WH_542 |  |  |  |  |  |  | 2 | 0.4091 | 0.2026 to 0.6584 |  |  |  |
| Wild type |  |  |  |  |  |  | 2 | 0.0165 | -0.0728 to 0.1398 |  |  |  |
| WH-711 |  |  |  |  |  |  |  |  |  | 2 | 0.3036 | 0.1236 to 0.4785 |
| Xinong 9871 |  |  |  |  |  |  | 8 | 0.0106 | 0.0011 to 0.027 |  |  |  |
| Yan 995 |  |  |  |  |  |  | 3 | 0.0253 | 0.0064 to 0.0814 |  |  |  |

The negative bootstrap values indicate no difference between melatonin and control. The ln*RR*_++_ is the response ratio, and N is the number of observations for comparison.

Table S4. Response ratio of melatonin compared to control with bootstrapped 95% confidence interval on chlorophyll content, chlorophyll a, chlorophyll b, and carotenoid for different wheat varieties.

|  | Chlorophyll content | | | Chlorophyll a | | | Chlorophyll b | | | Carotenoid | | | Chlorophyll SPAD | | |
| --- | --- | --- | --- | --- | --- | --- | --- | --- | --- | --- | --- | --- | --- | --- | --- |
| Varieties | N | RR | Bootstrap confidence intervals | N | RR | Bootstrap confidence intervals | N | RR | Bootstrap confidence intervals | N | RR | Bootstrap confidence intervals | N | RR | Bootstrap confidence intervals |
| Aikang58 | 2 | 0.1197 | 0.0345 to 0.2032 |  |  |  |  |  |  |  |  |  |  |  |  |
| Akbar-20 | 2 | 0.1271 | 0.1244 to 0.1299 | 2 | 0.1029 | 0.0665 to 0.138 | 2 | 0.2396 | 0.0983 to 0.3821 |  |  |  |  |  |  |
| Bezostaja-1 | 10 | 0.1973 | 0.022 to 0.4247 |  |  |  |  |  |  | 10 | 0.2365 | 0.0668 to 0.45 |  |  |  |
| Chuannong_19 | 6 | 0.0356 | -0.0384 to 0.1556 | 6 | 0.0334 | -0.0325 to 0.1386 | 6 | 0.0438 | -0.0432 to 0.1753 |  |  |  |  |  |  |
| Faisalabad-2008 |  |  |  | 4 | 0.5355 | 0.3711 to 0.6776 | 4 | 0.268 | 0.107 to 0.5485 | 4 | -0.0587 | -0.115 to 0.0172 |  |  |  |
| Giza_168 |  |  |  | 2 | 0.6183 | 0.516 to 0.9019 | 2 | 0.6805 | 0.5368 to 0.821 |  |  |  |  |  |  |
| GOAL |  |  |  |  |  |  |  |  |  |  |  |  | 2 | 0.2626 | 0.0637 to 0.5748 |
| Hengguan35 |  |  |  |  |  |  |  |  |  |  |  |  | 2 | -0.1599 | -0.1926 to -0.1272 |
| Jimai 22 |  |  |  |  |  |  |  |  |  |  |  |  | 2 | 0.0578 | -0.0015 to 0.117 |
| Lasani-2008) | 16 | 0.3279 | 0.2696 to 0.3807 | 22 | 0.288 | 0.2267 to 0.3539 | 22 | 0.2848 | 0.2406 to 0.3296 | 22 | 0.2455 | 0.1839 to 0.3084 |  |  |  |
| Lianmai_6 | 2 | 0.0267 | 0.0085 to 0.0449 | 2 | 0.0481 | 0.0409 to 0.055 |  |  |  |  |  |  |  |  |  |
| Lianmai_7 | 6 | 0.468 | 0.1507 to 0.8519 |  |  |  |  |  |  |  |  |  |  |  |  |
| Pandas | 12 | 0.1027 | 0.0468 to 0.1623 |  |  |  |  |  |  |  |  |  |  |  |  |
| Shannong_33 | 4 | 0.3907 | 0.1881 to 0.6601 |  |  |  | 4 | 0.4021 | 0.2122 to 0.6459 | 4 | 0.3801 | 0.1514 to 0.6219 |  |  |  |
| Sids_14 |  |  |  | 3 | 0.1769 | 0.0916 to 0.2763 | 3 | 0.249 | 0.1431 to 0.3594 | 3 | 0.293 | 0.1773 to 0.429 |  |  |  |
| Ujala-2016 |  |  |  | 4 | 0.7287 | 0.4503 to 1.3386 | 4 | 0.269 | 0.065 to 0.6423 | 4 | -0.0777 | -0.1916 to -0.0096 |  |  |  |
| WH 542 |  |  |  |  |  |  |  |  |  |  |  |  | 2 | 0.489 | 0.2892 to 0.8044 |
| Xi_Aimai-1 | 2 | -0.2025 | -0.4834 to 0.0671 |  |  |  |  |  |  |  |  |  |  |  |  |
| Xinong 9871 |  |  |  |  |  |  |  |  |  |  |  |  | 8 | 0.0397 | 0.0119 to 0.0987 |
| Yangmai-5 | 2 | -0.1418 | -0.2585 to -0.0305 |  |  |  |  |  |  |  |  |  |  |  |  |
| Yunong_703 |  |  |  | 2 | 0.1483 | 0.0051 to 0.293 | 2 | 0.1521 | 0.0121 to 0.2907 |  |  |  |  |  |  |
| Yangmai 18 |  |  |  |  |  |  |  |  |  |  |  |  | 8 | 0.0226 | 0.0124 to 0.0347 |

The negative bootstrap values indicate no difference between melatonin and control. The ln*RR*_++_ is the response ratio, and N is the number of observations for comparison.
